# Between the sea and the sky: A social practice investigation into health behaviours during cruise travel

**DOI:** 10.1002/hpja.593

**Published:** 2022-03-21

**Authors:** Olaf Werder, Kate Holland, Theaanna Kiaos, Mark J. Ferson

**Affiliations:** ^1^ University of Sydney Sydney Australia; ^2^ 2234 University of Canberra Canberra Australia; ^3^ 7788 Macquarie University Sydney Australia; ^4^ South Eastern Sydney Local Health District UNSW Medicine Randwick Australia

**Keywords:** health attitude, health communication, narrative interview, risk behaviour, social practice

## Abstract

**Issue addressed:**

The COVID‐19 pandemic has highlighted organised cruise holidays as perfect incubators for microbiological infections due to the constant socialising within closed spaces. Little is known about people's health behaviours and perceptions during cruise holidays.

**Methods:**

Narrative group interviews and respondent photo diary exercises were conducted with families (n = 25) residing in different areas across metropolitan NSW, Australia. Guided by a social practice theoretical approach we undertook a thematic analysis that identifies reasons for choosing a cruise, health considerations and behaviours in relation to cruise travel and awareness of official cruise health information.

**Results:**

Cruise travel included a licence to abandon cautious behaviours, reinforced by confidence in the cruise organiser's risk management ability. Health concerns were not a high priority for participants and were mainly understood in terms of eating healthy, modest exercise, managing seasickness and having adequate supplies of medications. Awareness of official cruise health and risk information was largely non‐existent.

**Conclusion:**

Understanding how travel health practices emerge and are likely to be modifiable produces health‐promoting awareness and intervention efforts that recognise and link with people's ideas about cruise holidays as times of fun, leisure, relaxation, without interfering with or imposing on them.

**So what?:**

This study highlights the importance of developing health communication and promotion strategies that are responsive to the interconnected meanings, competencies and materials that have a bearing on how cruise travellers understand and enact health‐related behaviours in preparation for and during a cruise holiday.

## 1 INTRODUCTION

Until the start of the COVID‐19 pandemic, cruise ship holidays were an exponentially growing popular mode of travel and attracted large numbers of people from all over the world. In Australia, cruising ranked among the fastest growing tourism sectors, with 5.8% of the population cruising by 2018.^1^ Since cruise ships resemble small towns, carrying up to 9000 people, public health challenges associated with cruising are substantial, exacerbated by semi‐confined spaces, shared facilities, and perceptions and claims of safe and carefree environments.

The complexity and variety of illnesses on cruise ships has shifted over the past three decades, moving from food‐borne bacteria outbreaks to viral outbreaks, caused by the norovirus – the leading cause of human acute viral gastroenteritis^2^, ^3^ – and, most recently, the lethal coronavirus strain. These health risks have the potential to develop into health‐related crises, which can impact the personal health and wellbeing of passengers, crewmembers, and even local residents.^4^, ^5^

Despite cruise ships being perfect incubators for microbiological infections from viruses and bacteria due to the constant socialising within closed spaces, the fact that many older (retired) people, who often already have certain health conditions like cruise travel, and the popularity of group activities on cruise ships, most passengers and crewmembers are largely unconcerned by certain health issues and pay little attention to what public health authorities call “healthy cruising” behaviour.^4^, ^6^

This is not for the lack of trying. Government health agencies have developed digital and traditional information and education in the past four decades. For instance, in the United States, the Centres for Disease Control (CDC) started their Vessel Sanitation Program (VSP) in the 1970s.^2^ With Sydney being Australia's largest cruise gateway, the responsible authority – the South Eastern Sydney Public Health Unit (SESPHU), an agency within one of the 15 local health districts of NSW Health – began their cruise ship health surveillance and inspection program in 1992. As part of that, it has absorbed a passenger information and education role, which runs under the header “Your guide to healthy cruising.”^7^ It is a short electronic pamphlet available through their website that provides a dot point list of things to do in preparation for a cruise (see your doctor, ensure you have enough of your regular medications, organise insurance, check what public health issues your destinations may have) and during the cruise (wash hands and use sanitiser, use your own cabin bathrooms, avoid uncooked meals, fill your water bottle on board, use mosquito repellents and seek medical attention if you become unwell).

Not unlike any other attempt to communicate disease prevention messages to the public, travel health communication exists within a complex social context. People are exposed to a range of health information and advice in their everyday lives. Much of this may not be personally relevant and thus is easily ignored. But even when health communication and promotion efforts are relevant, there may be a range of reasons why they do not connect with people. Top‐down communication models might attribute this disconnect to ignorance (deficit) or even a blatant refusal to comply. However, we suggest the how and why of accessing and embracing health information, particularly in pursuit of some form of behaviour change, is a much more complex and complicated matter. Persuading people to change their existing behaviours isn't all that easy, especially if it requires the abandonment of a behaviour that has been constructed because of ongoing communicative interactions at varying intensities with expert, social and/or lay sources.^8^, ^9^, ^10^

If we accept that people's general beliefs about health derive partly from the degree to which people feel they have control over their daily lives,^11^ it is important to understand that people interpret and enact ‘healthy’ behaviours in the context of their attempts to achieve or preserve their own living conditions and circumstances. From this perspective, official health information is interpreted through the lens of what it means for one's current life, whether one can do what is being asked or required of them and if the infrastructure and materials enabling them to do that are available.^10^ Without understanding these personal conversions, the many health messages that come with ‘best practice’ suggestions (do this, don't do that) can easily lead to a clash of viewpoints that may not only irritate or alienate the very groups that are targeted,^12^ but also have great potential to be perceived as paternalistic infringements on one's autonomy by the message sender. While information and education approaches to changing people's attitudes and behaviours remain an important component of travel health promotions and programs, we argue effective communication requires more than the mere distribution of information or risk warnings.^13^

The present study draws on a social practice framework developed by Shove et al^14^ to investigate people's health understandings and behaviours in preparation for and during a cruise holiday. Social practice theory shifts the focus of inquiry away from the attitudes, discrete behaviours, and choices of individuals to socially embedded practices that are shaped by one's habitus and socialisation.^10^, ^14^, ^15^ Practices are seen as comprising several interconnected elements. Although various configurations have been proposed, the model proposed by Shove et al,^14^ devising an interplay between competence (the skill to engage in a practice), material (the availability of required tools or infrastructures, eg access to information), and meaning (understandings shared by a group about the practice), has great potential when targeting behaviour change by intervention campaigns/policies.^16^ To visualise how these influence factors correlate, see the structure of the practice elements from Shove et al,^14^ shown in Appendix A. While social practice theory has yet to have a major influence on mainstream health communication research and prevention campaigns, sociologists of health and illness have provided valuable insights.^17^

Health behaviour before and during cruise travel as a social practice is the basic unit of analysis, whereby cruise travellers are decentralised as carriers of this practice. This stance is in stark contrast to that in most pro‐health behaviour studies, where people are at the centre of analysis and their satisfaction, motivation, and attitude (toward the specific healthy behaviour that is studied) are primarily examined.^18^ Translating this to our focus on cruise travel, our aim was to understand travel health perceptions and behaviours in the context of the meanings, competences and materials associated with cruise travel. The application of social practice theory thus affords a middle path to reinterpret the relationship between agency and structure, and accordingly, could avoid both the trap of methodological individualism and that of holism.^19^ Those will provide guidance on how industry and authorities can develop more effective strategies. This includes paying attention to how current intervention messages connect or resonate with cruise travel and travel preparation practices. To arrive at this, we probed participants’ ideas of what (own behaviours and external circumstances) makes the cruise fun and enjoyable as well as what (own behaviours and external circumstances) makes the cruise safe and healthy.

We employed a variety of participatory methodologies to capture embodiments of social practices in their context.^20^, ^21^ This included participants providing their own photographs – known as photo elicitation technique^22^, ^23^ – about fun and health during the cruise. These allowed participants to reconstruct how they were feeling at the time the photograph was taken. These were later included in ethnographic, narrative interviews to elicit family practices regarding their travel.^24^, ^25^ This type of interviewing allowed participants to tell their stories in the way they choose, using their photographs as inputs and mnemonic devices to recall experiences.

## 2 METHODS

### 2.1 Ethics approval

Ethics approval was obtained from the South Eastern Sydney Local Health District Human Research Ethics Committee. Prior to their cruise and each interview, participants consented for their interview to be digitally recorded for later transcription and analysis. No adverse events occurred, and only four people were withdrawn from the study due to changes in their eligibility.

### 2.2 Study design

Having an exploratory nature, this project used a qualitative ethnographic approach^24^, ^26^ to gain insights into passengers’ health practices and perceptions during cruise holidays. After an initial informational take‐in interview, participants had to participate in a self‐monitoring photo‐diary exercise during their cruise.^27^ This activity involved each family member individually taking two sets of pictures with their mobile device: the first one illustrating their idea about having fun on a cruise holiday, the second one indicating what their idea of healthy cruising behaviour means.

After the cruise, an ethnographic interview with the family was conducted to reflect on the photo diary exercise (pictures were shown on a laptop for reference) and to explore and explain their experiences and opinions on and about the cruise. Questions included current prevention behaviour and steps taken to maintain one's health in relation to usual behaviour prior to and during a cruise.

### 2.3 Data collection

#### 2.3.1 Recruitment and participation

A self‐selected sample of eleven family units (families of two or more), who were residents in the greater Sydney area and had booked on a cruise during the data collection timeframe were recruited between July‐October 2019. All participants were recruited after responding to project announcements in social media (Facebook, Twitter) and on independent cruising and the health partner's websites.

Families who responded to the recruitment call received a letter with a brief introduction to the study, including information on the diary task. A week later, they were telephoned by the interviewer to obtain consent and arrange for an initial intake interview. The interview, with a mean duration of 30‐45 minutes, contained questions on sociodemographic variables, social roles, travel plans, as well as a Q&A session for follow‐up questions on the in‐cruise photo diary. Participants also received an instruction sheet about what photos to take and how to save them and a date for the post‐cruise follow‐up interview was discussed.

Within a week after their cruise date, participants were telephoned again by the interviewer to finalise the interview and obtain consent for video recording it. The ethnographic interview (see Appendix B), with a mean of 90 minutes, focused on descriptive, structural and contrast questions^28^ about fun during cruising and health concerns during cruising, intended to produce narratives about socially embedded experiences.

#### 2.3.2 Participation

Of the participants who took part in the initial interview, 85% completed the full cycle of interviews and photo diaries. Only 5% of the people who contacted us withdrew after learning about the protocol; another 10% had to drop out for personal reasons. In the end, we obtained 10 group interviews and a total of 35 photo sets.

### 2.4 Data analysis and management

Two researchers watched and analysed the recorded interviews and photo sets and compared notes to identify themes and patterns.^26^ The two researchers reviewed coding structures and themes, and discrepancies were resolved through consensus to ensure adequate agreement and refine themes. Data saturation was achieved, such that no new themes or information were being identified. Three overarching themes are discussed: Reasons for choosing a cruise holiday; Health considerations and behaviours on the cruise; and Knowledge of official cruise health information efforts.

## 3 RESULTS

### 3.1 Group characteristics

A total of ten group interviews were obtained, comprising 30 individuals, of whom 7 were children <18 years and 19 were adults. Of the ten family units, 70% were similar‐aged couples and 30% were families traveling with children. There was an approximate balanced gender profile (55% female). Of the 30 adults, 40% were under 40 years of age with another 20% each distributed among 40‐49‐year‐olds and 50+ year‐olds. The adult participants worked across a variety of occupations from nursing, administrative/office jobs, trades, and professional jobs. Eight groups travelled on Pacific cruises (primarily Pacific Island destinations), one group travelled further afield on the Pacific (stops in China and western North America), and the final group travelled on European river and Atlantic cruises. Twelve participants travelled in package tours as part of an event.

### 3.2 Reasons for choosing a cruise holiday

The meanings people attach to a cruise holiday are important to consider. It became clear in the various interviews that participants had chosen a cruise holiday as it provides an environment where they “*don't have to worry about anything*” (male, 20s) because “*the cruise company takes care of me*” (female, 30s). In fact, each group displayed a high level of trust in the cruise staff as the company is big enough and prepared enough so staff can act when required to cover any eventuality. This in turn allowed passengers to focus on the reasons they chose to cruise in the first place.

#### 3.2.1 Cruising as stress relief

One of the most cited reasons for taking a cruise was to get away from it all from the start because the travel time to any destination would be already part of the holiday, if not the holiday itself. Two typical answers to the question why they chose a cruise were “*I could be sitting at work right now but instead I’m sitting on a boat with a cocktail in my hand*” (male, 20s), and “*We like to cruise as we don't have to unpack all the time and can walk around*” (female, 70s). This combination of relaxation and inspiration (via on‐board entertainment and activities) was subsequently also among the most chosen photographic interpretations of having fun (see Appendix C).

#### 3.2.2 Cruising as reward

Equally frequent were mentions of indulgence and letting one's (health) guard down to reward oneself, well summarised by the following statement: “*I reckon we ate too much ice cream – oh well, it's our holiday*” (female, 20s). Similarly, while acknowledging overeating as an unhealthy behaviour facilitated by the buffet style meal packages, being considered a reward, it was also rationalised within the holiday context and explained as “under control”, as shown the following exchange:

Q: *Have there been moments where you thought you did not act very healthy?*

A (male, 20s): *Usually during mealtimes …*

Q: *How is that?*

A: *We bought the meal plan and since food was mostly buffet style, I often only stopped when I felt so bloated that I didn't know how to get back to the cabin*.

Q: *How did that make you feel?*

A: *Tired, but then again – it's the holidays, and we also walked a lot on the boat so I’ve got it under control*.

The notion of the holiday as a carefree time with the potential to win prizes or stand out in performances showed up repeatedly in the cruise fun photos, showing invariably scenes of people participating in live entertainment, gaming/gambling activities, groups frolicking on deck or in designated play zones, and close‐ups of all kinds of indulgence foods from cocktails to hamburgers and sweet desserts (see Appendix C). This narrative did routinely entail (or at least imply) a licence to abandon cautious behaviours whether they were financial (one interviewee talked about visiting the on‐board or destination location shops as retail therapy) or health‐related (another interviewee observed passengers starting to drink alcohol in the morning). A cruise holiday provides the opportunity to temporarily leave behind the humdrum of one's everyday life and, with this, ideas about what being healthy means are also adapted.

### 3.3 Health considerations and behaviours on the cruise

Health related considerations and planning in preparation for the cruise were limited though this did vary depending on the type of person, previous cruise experience and current health issues or those of family members. In general, health preparedness seemed to be an extension of everyday onshore practices (ie having Panadols in one's handbag or taking vitamins), with maybe the addition of bringing seasickness tablets and hand sanitisers.

#### 3.3.1 Understandings of healthy behaviour

In terms of people's preparations for and behaviour during cruise holidays, health considerations and concerns did not appear to be particularly prominent. Some downplayed any concern (“*I’m healthy, no need to prepare for my trip*,” female, 20s) while others had taken some steps to prepare (“*Be active, bring supplements*,” male, 30s; “*We bought travel insurance*,” female 50s). About 70% of the respondents also packed what they considered essential medical necessities. For some, this included preventative over‐the‐counter drugs and supplements, for others necessary prescription medication, and one couple enquired about vaccinations (“*Yeah, I checked on travel vaccinations with our GP before our cruise*” (male, 70s). A mother whose son had health issues investigated what type of doctors were on board and where the nearest hospitals were in case something happened to him on the cruise.

In general, discussing health as part of the cruising experience seemed for almost everyone a strange or unnecessary topic. It surprised us that the health‐related photos from the photo diary seemed reduced by all participants to healthy foods/beverages, sports activities and whatever tools and information the cruise ship made available for passengers. This trend continued in the free‐flowing interviews, where respondents when pressed to think about it, again reflected only on eating healthy, modest exercising and dealing with seasickness to describe their thoughts on healthy behaviour.

Given the paucity of concerns with health, passengers in this pre‐COVID timeframe could only think of preventing sea/motion sickness, sunburns, and dehydration as possible serious health scenarios. Contrast this with the official healthy cruising document,^7^ which does also include viral/bacterial risks, insect‐borne diseases, foodborne illnesses, behaviour when unwell, and social distancing.

In line with other studies,^29^, ^30^ people listed home remedies as a first solution. For example, comments about drinking plenty of water, washing hands, lying in bed when feeling sick, packing supplements, and aiming to eat healthy were recurring statements. In addition, our respondents felt adequately equipped for those events: “*I took Panadol, throat lozenges and seasickness pills with me*” (female, 30s). Aside from these occurrences, people did not seem to envision any major health crises, nor would be swayed to engage in more drastic responses should one happen; “*If I had heard of a health crisis before our trip, I would still go but pack more meds*” (male, 40s).

#### 3.3.2 Modelling from others

Reflecting on the above, we wanted to probe deeper into the influences on people's health‐related behaviours. Overall, they appear to be a mix of routine practices in everyday life, executing suggestions learnt from social media or word‐of‐mouth pre‐travel, and a herd mentality, following others’ behaviours.^31^, ^32^ For example, on the topic of seasickness almost every group had their own best practices on how to deal with it, should they get seasick. Some had heard that one ought to eat green apples, others claimed that it is useful to leave the TV on for distraction, a third group had “*spoken to a fellow traveller who said one should lie in bed but leave one foot on the ground*.” The consensus was that one ought to just wait it out in the cabin as there is not much one can do about it. While seasickness was an expected potential health threat, it appears there was less concern about its health impacts than about its interference with fun activities.

Modelling behaviour also works in the desired direction if the infrastructure is provided. In the case of hygiene practices, for example, just about everyone who was interviewed not only commented on the large numbers of sanitiser stations, mechanical no‐touch public bathroom doors, or sneeze guards at buffets, they also reported using them regularly. While this primarily impacts hygiene only, given the typical health risks onboard a cruise ship, it appears that people were successfully guided in engaging in routine sanitising behaviour patterns via vicarious learning and mimicking others.^33^ Their confidence in the cleanliness of the ship was also reinforced by the visibility of staff regularly cleaning.

#### 3.3.3 Awareness of health services onboard

Participants’ awareness of available health services on the cruise was varied. Whereas most respondents were very aware of evacuation routes and procedures in case of a maritime disaster, many were less aware where to locate the medical clinic on the ship, if they knew about it at all: “*When we walked around one day, we accidentally saw the medical centre sign; first time I saw that on a ship*” (male, 30s). Exploring this service further, most people referred to stories being told by their cruise directors that the ship has health services for small medical issues all the way to a morgue. Some suggested the medical centre was difficult to find on purpose, and there seemed to be little awareness of its staffing. Most also believed or had heard through the grapevine that the costs of using health and pharmacy services were exorbitant, and thus they displayed an unwillingness to use them: “*If I get really sick, I suppose I go to a doctor when we dock someplace*” (female, 40s). It appears that any concern that a lack of knowledge about available health services could arouse is buffered by a reigning norm that there is nothing to worry about in terms of serious illness.^34^, ^35^

In fact, over 80% of interviewees merged on the prevalent perception that “*the cruise ship is prepared, they cover everything*.” Participants expressed high confidence in the ability of cruise staff to manage sickness and injury on board and gave examples of observing staff helping passengers who were unwell or injured. As a result, aside from following one's everyday social or health practices or preparations, no special care is required.

### 3.4 Knowledge of official cruise health information efforts

Our results indicate that participants were totally unaware of government prevention efforts and sources about healthy cruising behaviours, although they readily exist as pamphlets and on the internet.^7^ In light of the findings already discussed about people not envisaging serious health scenarios during their cruise holidays, this is not surprising. What they did identify when asked about travel health and risks is informative.

#### 3.4.1 Ideas and sources of information about travel health risks

People struggled to conceive of what health guides for traveling or, more specifically, cruise travel would assist them with. When pressed for a source of this kind of information, many related this to the typical travel risks mentioned by the ‘Smarttraveller.gov.au’ site of the Australian Department of Foreign Affairs and Trade. Others mentioned tips to be environmentally aware and not pollute or toss rubbish. A few respondents thought of sun protection advice (along the lines of the Australian Cancer Council's *Slip, Slop, Slap* campaign) or exotic disease travel vaccination awareness efforts. Yet, besides intuitively engaging in recommended preparations and healthy routine practices (personal hygiene, packing medicine), they trusted the cruise company (and their travel agency if booked through one) to take care of them in case of illness and provide required information and steps to take prior to the travel date.^36^

Unlike other risks of travel (geopolitical, crime), health information was not regarded as a priority concern. Judging from people's statements, making it so would require a collaborative and proactive effort between government and travel companies: *“[Health] is the host's responsibility; they are the host when I am onboard*” (female, 50s).

We also asked whether increasing a social media presence would assist a health entity with their cruise health awareness efforts. Yet the usefulness of social/digital media was unclear, with some seeing a benefit if in the right domains and others preferring sources more proximal to travel preparation and commencement (doctors’ waiting rooms, check‐in process, ticket stub short messages). Overall, people do not seem to like to be “pushed” to a specific digital media environment but preferred to proactively seek out information they deem relevant or interesting. Most mentioned Facebook (especially cruise travel groups), cruise line and cruise traveller website links or blurbs, and direct e‐mail or postal mail information from cruise organisers that would discuss health among other relevant information (this one applies once someone has booked a trip only). One young woman – part of a travelling young couple – told a longer story of a work colleague alerting her to a Facebook cruise travel group, where she then found some interesting information of all kinds, among them some tips for what to pack for staying healthy.

Asked about what they would do if in charge of an entity responsible for providing cruise health information, respondents had a number of ideas ranging from ‘nothing because no need’ to Facebook chat groups, short SMS messages with links to more details, image‐heavy messaging (less text), community‐based efforts, involve travel/cruise companies, make information very easy to find no matter what it is, onboard health officers, offer health insurance to avoid non‐use of services as a result of affordability, and signs in local doctors’ offices. We may add that his was age‐dependent with younger travellers opting primarily for digital/electronic engagements, and older travellers (especially retired ones) opting for direct human contact and service offers.

That said, it appears to us that it is less about creating materials with the maximum amount of appropriate health information (or facts of risks) and distributing that through some channel. After all, much of the health information provided in those materials was something people could also come up with when prompted or did habitually on their own. Looking through the lens of social practice, looking after one's health during travel or cruising exists as an as‐needed concern,^37^, ^38^ summarised by the following commentary: “*I really don't look for anything before a cruise. This is an individual problem, so I read something when I need it. Otherwise, I have no curiosity*” (male, 70s). In other words, to increase people's curiosity requires moving disease prevention awareness and precaution closer to routine practices in both content creation and content delivery.

## 4 DISCUSSION

To summarise our findings and illustrate them visually, we revisit the Shove et al^14^ model and apply it to our healthy cruising behaviour context (Appendix A). As we can see, the provision of safety/health information and support of health‐aware skills are relevant factors, but they are both reliant upon the meaning making an individual traveller deduces from it and/or wishes to engage with it. In short, the effectiveness of advocacy and enablement are dependent upon community participation and acceptance when it comes to promotional activities. Agencies responsible for providing cruise health information to prospective travellers need to consider a variety of factors. We will come back to this below.

The responsible health agencies for cruise ports and travel typically strive to provide sufficient information and communication efforts regarding infectious disease risks toward prospective and actual travellers for those to engage in healthy cruise behaviours and stay healthy. Yet, we found that the pre‐ and during‐cruise health behaviours displayed by our respondents echo basic behavioural patterns (eg handwashing, packing necessary medication, drinking water, wearing hat/sunscreen) that seem common‐sensical and are known to and executed by our respondents, who claim to not know about the materials published by public health agencies. How then can these findings inform the health promotion efforts of entities responsible for providing cruise health advice to prospective travellers?

This qualitative ethnographic study of the health perceptions and behaviours of cruise travellers in preparation for and during their cruise holiday is an important first step to inform cruise health promotion efforts in accordance with the Ottawa Charter and strategies that enable, mediate and advocate, enlisting the participation of a variety of sectors.^39^ A cruise ship is in many ways a unique setting in which cruise companies, responsible government agencies and individual cruisers each have important roles to play in ensuring disease prevention measures and positive health outcomes for cruise travellers and crew alike. The COVID‐19 pandemic has certainly highlighted that it is in the interests of all concerned for the cruise environment to be a safe and healthy one and for the mechanisms that regulate and monitor disease outbreaks, for example, to be transparent and coordinated. Thus, while we have focused in this study on individual cruise travellers, its findings reflect and must be understood within this larger context. Cruise companies have a clear interest in promoting cruises as a fun, relaxing, entertaining holiday option and, along with travellers, a clear interest in ensuring that these aspirations are realised in conjunction with, rather than at the expense of, health and safety.

Applying a social practice framework^14^ we note that most people display the required competence to safeguard against unforeseen illnesses and health risks (many packed seasickness pills, everyday over‐the‐counter medication, sunscreen, and regularly used provided sanitiser stations). Some of the ‘materials’ to enable and encourage health protective behaviours existed (eg the above‐mentioned sanitiser stations), while others were either perceived as unavailable (specialist medical care) or regarded as unaffordable (on‐board pharmacy products). In terms of promoting awareness of healthy cruise behaviours, it is the meaning dimension of cruising as a social practice that health agencies need to better understand, particularly as going on a cruise is associated with many health promoting activities (eg relieving stress). Most participants displayed a sense that worrying about one's health is not an enjoyable or beneficial attitude during a fun time, especially when there is an expectation that cruise companies will be on top of any possible health risk. A cruise holiday primarily means fun and relaxation, which perhaps explains why serious health concerns were not at the forefront of people's minds and, when prompted, they tended to think about health through the lens of their everyday routine health‐related practices and their general cruise activities.

To address health promotion for future cruisers one first needs to understand the position of that group. Our results help to explain inadequacies of current approaches where factual data points and suggested behaviours are dot‐points, listed on the government website. We do not deny that top‐down messaging tone may work well enough when people are in a heightened state of concern and actively seek out information^40^ (eg COVID‐19 messaging may be a case in point here), as it is conceivable that a viral pandemic (like the current one) will instil in the average person a bigger drive to seek out and consult government and related information sources on health protective behaviours.

However, if health concerns are not present or are superseded by other beliefs and behavioural orientations, top‐down information dissemination is likely to have limited effectiveness, even if multiple channels (traditional and digital media) are used. It reminds one of the proverbial “field of dreams” concept (build it and they will come) where the simple dissemination of a message ensures engagement and adequate behavioural responses. Our study shows that even if such information is available – in whatever form and via whatever channel – people still need to be motivated to seek it out, which they are not predisposed to doing because ‘health’ is not at the forefront of their minds when preparing for and enjoying a cruise holiday. Participants did not report a great deal of concern or preparedness in relation to seeking out health information prior to their cruise.

What a social practice approach then contributes for the development of health promotion strategies is to reflect on message content and delivery not so much from a goal‐orientation or risk avoidance purpose but from the reception or receptivity of an audience amid their everyday routines and distractions. In this context, efforts to promote health as a priority concern for cruise travellers would do well to pay attention and seek to link into, without interfering with or imposing on, people's ideas about cruise holidays as times of fun, leisure, relaxation, which are also health promoting. Aside from more radical ideas of consumer consulting panels co‐deciding on messages and distribution, a possible avenue for that would be an integration of those messages in travellers’ everyday practices, including typical media engagements, or proper partnership selection to achieve a 360‐degree approach.

### 4.1 Strengths and limitations

Like other studies of this nature, selection bias is a limitation of this study. Those who self‐selected to participate were more likely to be interested in talking about cruise travel and health. While we aimed to recruit participant groups of all sizes, most participants in the study were couples. Moreover, the data in our analysis relied upon self‐re‐ports in a group interview setting. It is therefore susceptible to social desirability bias, which may have led to over‐reporting of healthy behaviours.

We can highlight two key points from our findings for further discussion: (1) understanding how travel health practices relate to other health‐supporting or ‐blocking activities of everyday life offer insights into how, why, where, when and with whom (un)healthy cruising behaviours occur; (2) applying these insights provides public health authorities an avenue to connect their aims better with people's everyday lives and routines.

Overall, the study provides one of the first in‐depth explorations of patterns of cruise behaviours, weighed against perceptions and knowledge of adverse health consequences of cruising. These findings provide a foundation for larger studies to test promotional efforts and to guide interventions aimed at aligning travel holiday behaviours with health‐preserving practices and point to the value of understanding routine travel practices, and subsequent engagement and reception of prevention messaging by the cruising public.

## 5 CONCLUSIONS

A central point is that government health promotion on this issue is driven by a linear concept espoused by health psychology of behaviour being a direct result of variations in preceding cues, such as norms, influential cues on attitudes and the like. Subsequently, as Cohn has argued,^17^ “[*t*]*he social, affective, material and interrelational features of human activity are effectively eliminated, as behaviour becomes viewed as an outcome of the individual and determined only by such things as motives, intentions and the subjective reception of norms and cues*.”

Although the public health impact of cruise travel could be regarded as among the less crucial items on a government public health agenda, the sudden impact, and catastrophic consequences of the COVID‐19 pandemic on ocean cruising are an extreme demonstration of its importance. Once cruising resumes, it will be necessary to reconsider cruise passenger practices, and a health or social practice perspective provides context and a better understanding of the essential complexity of human behaviour. It offers prevention and awareness campaigns a different approach from the usual causal risk‐response model (*this* risk requires *that* individual behaviour) but includes the variety of social factors that drive practice. This may also apply to other contexts.

Studying how travel and travel‐preparation practices for cruise holidays fit with practices in other travel modes as well as in domains such as work, family and leisure will help us understand how interventions alter practices by changing elements (using nudging approaches), disrupting connections between practice elements, or changing the relationship between those that compete for time. For example, what health risks of holidaymaking mentioned in information pamphlets coincide with cruise travellers’ own definitions of what staying healthy means? Will current practices persist or continue in a slightly altered form (eg visit a doctor prior to travel)? Will people just move to other forms of travel as a result? How do different population segments respond to the same message? Is there a benefit to early or separate communication versus point‐of‐sale connected messaging (eg during ticket purchase, while coming aboard)? The processes mentioned in these questions reveal the complexity and interconnectedness of behavioural practices that are not only dynamic but a result of lived experiences and social influences.^41^, ^42^

The insights obtained via a social practice framework consider how healthy cruising policies and population‐level interventions or awareness campaigns might differentially affect different travel practices. The practice framework we applied indicated that the culturally ingrained travel practices of cruise travellers are influenced by the availability of visible hygiene stations and what risks, or health concerns are envisioned to need preparation. This suggests that any relevant cruise health information could be connected to the cleanliness and quality assurance of cruise lines, triggering further health decisions.

In closing, the development of innovative and creative health communication and promotion strategies that are responsive to the interconnected meanings, competences and materials can have a bearing on how cruise travellers understand and enact health‐related behaviours in preparation for and during a cruise holiday.^43^

## CONFLICT OF INTEREST

The authors declare no conflict of interest.

## ETHICS APPROVAL

Thank you for submitting the following Human Research Ethics Application (HREA) for HREC review; 2018/ETH00657. This project was considered by the South Eastern Sydney Local Health District HREC LNR Committee at its meeting held on 02/04/2019 and was determined to meet the requirements of the National Statement on Ethical Conduct in Human Research (2007). This project has been approved to be conducted at the following sites: South Eastern Sydney Local Health District (Site).

## Figures and Tables

**Figure:**
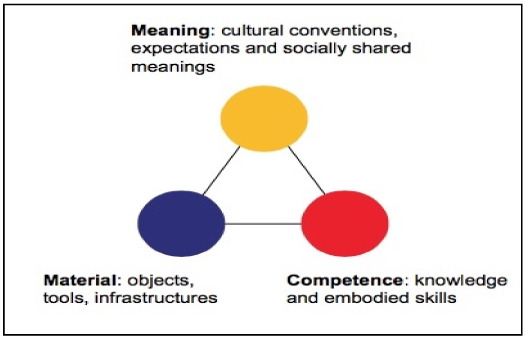
Social practice elements (adapted from Shove et al., 2012)

**Figure:**
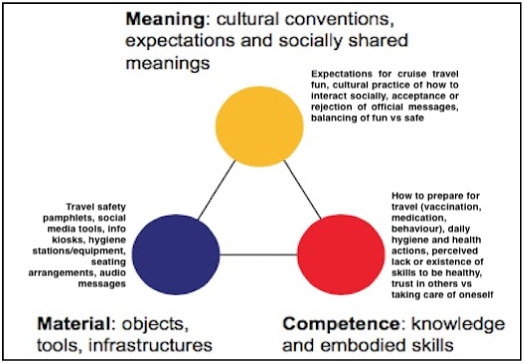
Application of Shove et al. (2012) for healthy cruising behaviour

## APPENDIX B

### Interview questions

**(I) Personal characteristics**

**(II) Travel habits**

1. What were the main reasons you chose a cruise for your holidays?
2. How do you plan and prepare for your cruise?
3. Do you usually worry about your personal/family's health when going on a cruise?
4. Do you check for health‐related information in preparation for cruising – if so, how do you go about finding information?
5. Have you been able to find sufficient health information to satisfy your needs?
  - *Probe to see if they could provide a specific example*

**(III) Cruise health behaviour**

1. Have you ever heard of or witnessed anyone getting sick during a cruise?
  - Did this change your subsequent cruise travel practices? If so, in what ways?
2. What would you do if you started feeling unwell during a cruise?
  - Where would you go?
  - Can you think of any reasons why you would not take action if you felt unwell?
3. Do you think a cruise line is well equipped to handle people getting ill?
4. How would you describe healthy cruise behaviour?
5. Do you think that people should plan for possible illnesses? How so? (OR) Why not?

**(IV) Health awareness/change efforts**

1. Are you aware of any government entity trying to educate or assist with health issues for international travel or especially cruise passengers?
  - (If yes) What kind of story about healthy behaviour do you think this group talks about in their materials?
2. Based on your cruise travel experiences, can you think of any information or resources that would have been useful to you in managing your health and wellbeing?
3. If you were in charge of an Australian or local travel health government group, what would you want to talk about and how would you try to reach people like yourself?
4. Have you ever actively used digital media/mobile apps that discuss health care while cruising or traveling in general? Can you name a few?
  - In the context of cruise health, what kind of information do you usually find digital media very useful for?
  - Alternatively, what kind of information do you need but cannot find in currently available sites or mobile apps of this kind?

## APPENDIX C

### Photo diary results

**(1) Fun Themes**

**(2) Health Themes**
